# Supplementary figures and images for: Psychological mechanisms of offset analgesia: The effect of expectancy manipulation
Source: PLoS One. 2023 Jan 17;18(1):e0280579. doi: 10.1371/journal.pone.0280579 (PMC9844857; doi:10.1371/journal.pone.0280579)

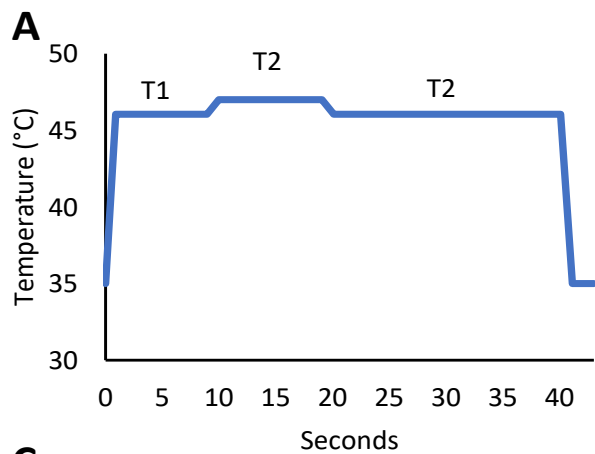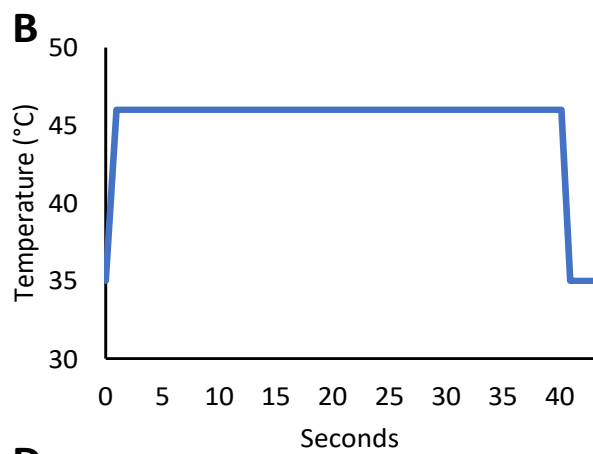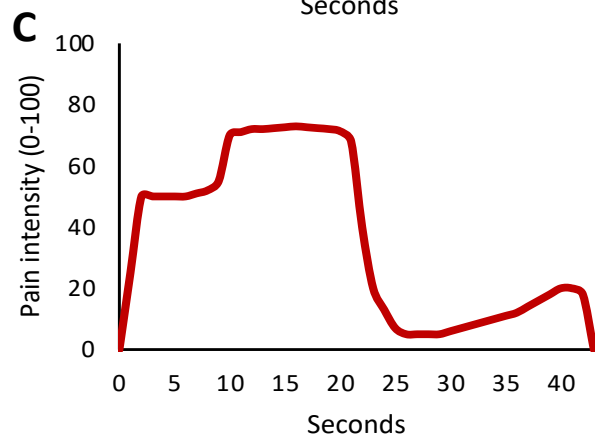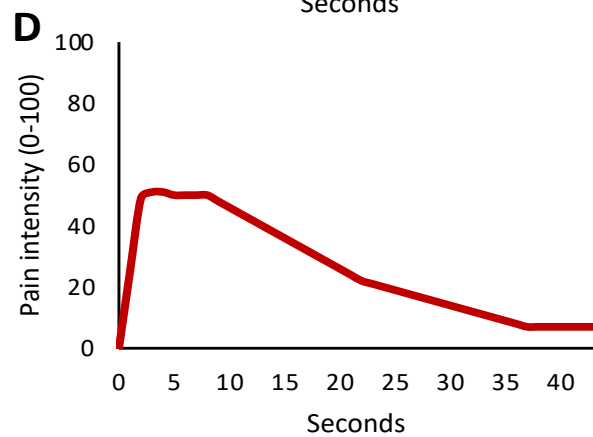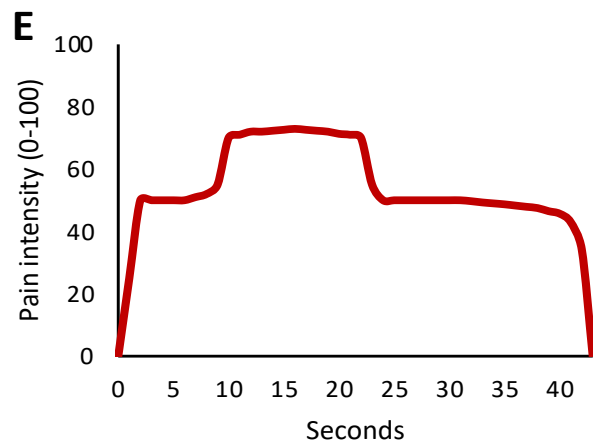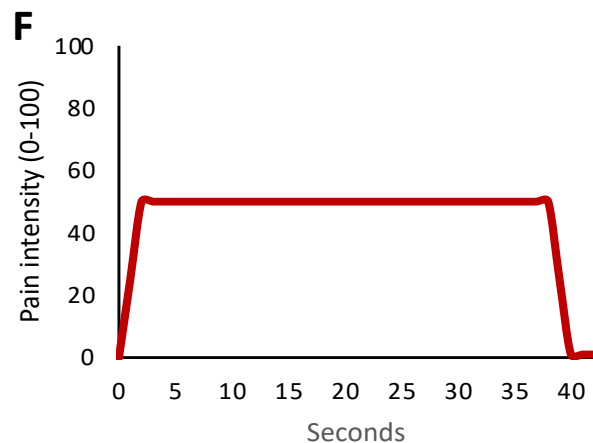

Supplement: S1 Fig — Heat stimuli within the Offset Trial (A): T1 interval (0–9 sec) at 46°C, T2 interval (10–19 sec) at 47°C, T3 interval (20–40 sec) at 46°C. Heat stimuli within the Constant Trial (B): constant at 46°C; suggestion figures of the hypoalgesic group during the Offset Trial (C), pain perception first increases to a level of 50/100, then to 70/100 and drops sharply in the last seconds to an almost non-painful level (approx. 5/100); during the Constant Trial (D), pain perception starts at a level of 50/100 and then slowly and constantly decreases; suggestion images of the hyperalgesic group: during the Offset Trial (E), pain perception first increases to a level of 50/100, then to 70/100 and finally to 50/100 again; during the Constant Trial (F), pain perception remains constant at a level of 50/100. (PDF) [file pone.0280579.s001.pdf]
