# Supplementary material for: Psychological mechanisms of offset analgesia: The effect of expectancy manipulation
Source: PLoS One. 2023 Jan 17;18(1):e0280579. doi: 10.1371/journal.pone.0280579 (PMC9844857; doi:10.1371/journal.pone.0280579)
Supplement: S2 File — (DOCX) [file pone.0280579.s003.docx]

**S3 File. Additional analytical method for offset analgesia.**

The pain response of the offset analgesia (OA%) was further analyzed using a method of analysis within parameters of the offset trial (OT). The OA% included the percentage difference between the maximum pain ratings for T2 (T2max) and minimum pain ratings for T3 (T3min) of the OT:

$OA\%=\frac{(T2max-T3min)}{T2max}*100\%$

The hypoalgesic group indicated an OA% of 90.3 (SD 14.0), whereas the hyperalgesic group and the control group had OA% values of 72.5 (SD 22.2) and 82.3 (SD 20.4), respectively. A significant difference for the factor “group” was found between the three groups in pain ratings at T3 (F_[2, 93]_ = 7.30, p = 0.001, η^2^_p_ = 0.14). Bonferroni-corrected post-hoc t-tests showed significantly different OA% in the hyperalgesic group than in both the hypoalgesic (p = 0.001) and control (p = 0.046) groups. In contrast, no significant difference was found between the hypoalgesic and the control group (p = 0.652).
