# Supplementary material for: Psychological mechanisms of offset analgesia: The effect of expectancy manipulation
Source: PLoS One. 2023 Jan 17;18(1):e0280579. doi: 10.1371/journal.pone.0280579 (PMC9844857; doi:10.1371/journal.pone.0280579)
Supplement: Attachment — Submitted filename: Plos One-22-22989.pdf [file pone.0280579.s006.pdf]

General
The current manuscript looks to examine the psychological mechanisms related to offset analgesia. While many mechanisms have been examined in relation to offset analgesia, the use of psychological interventions is a novel methods determining endogenous pain modulation. This study used suggestions to determine the degree of offset analgesia during two separate trials using an experimental heat stimulus. The study found that offset analgesia was present in all groups, regardless of the type of suggestion provided. While I feel the manuscript is very well written, there are some things that can be added to improve the overall quality.

COMMENTS

Introduction
Lines 64-71: you introduce the terms placebo and nocebo. You should define these terms for the reader. While placebo is fairly common, nocebo is less so and readers may not understand its intent.

Methods
Lines 98-103: It would help if you included the suggestions used in this section.

Lines 107-108: How did participants confirm they were otherwise healthy? Did you use a PAR-Q or some other questionnaire?

Lines 142-144: Explain in more detail how participants rated their perceived pain. Did they use a touchscreen or did they have a mouse? How often were ratings given?

Results
Line 230: Include a closed parathesis after 0.06 and include a space between that and ‘or’
