# Supplementary material for: Psychological mechanisms of offset analgesia: The effect of expectancy manipulation
Source: PLoS One. 2023 Jan 17;18(1):e0280579. doi: 10.1371/journal.pone.0280579 (PMC9844857; doi:10.1371/journal.pone.0280579)
Supplement: Attachment — Submitted filename: Response to Reviewers.docx [file pone.0280579.s008.docx]

**Journal Requirements:**

Please review your reference list to ensure that it is complete and correct. If you have cited papers that have been retracted, please include the rationale for doing so in the manuscript text, or remove these references and replace them with relevant current references. Any changes to the reference list should be mentioned in the rebuttal letter that accompanies your revised manuscript. If you need to cite a retracted article, indicate the article’s retracted status in the References list and also include a citation and full reference for the retraction notice.

*Response: Thank you for this comment. All cited papers were checked using The Retraction Watch Database. Minor errors were noticed following verification of the accuracy of the reference list. These have been corrected. All changes are marked in yellow.*

**Additional Editor Comments:**

The manuscript has substantially improved with the revisions and only is in need of minor revisions.  Specifically, as pointed out by Reviewer 2, it appears that the dataset shared does not include the complete dataset.  Also, the timeseries data plotted in Figures 2 and 4 does not appear in the dataset file. Please include a data dictionary for all of the variables.

*Response:* *Also thank you for this comment. The dataset has been revised. On the first page of the excel table (S4_File) you now find a clear data dictionary. The characteristics follow on the second page. This is followed by the pain ratings and EDA responses divided by the three groups. We believe that the dataset is now clear and concise.*

**Reviewer #1:**

The manuscript is substantially improved. Additionally, the letter response provided some excellent discourse which was much appreciated.

*Response: Thank you again for reviewing our manuscript. We are sure that your suggestions have improved the quality a tremendous amount.*

The one issue remaining is that the dataset shared does not include the complete dataset. The timeseries data plotted in Fig 2 and Fig 4 is not in the dataset file. Additionally, a data dictionary should be included for all variables. Prior to publication, please confirm supplemental files have the correct number within the file.

*Response: We have revised the dataset. The first page of the Excel spreadsheet (S4_File) now includes a straightforward data dictionary. Following on the second page are the characteristics of the study subjects. Next are the pain ratings and the EDA responses, divided by the three groups (hypoalgesic, hyperalgesic, control). We believe the data set is now clear and concise. We have also corrected the typo in the numbering of the supplemental files.*
